# Closure of the thoracic duct from the left-side access: A case report

**DOI:** 10.1097/MD.0000000000004552

**Published:** 2016-09-02

**Authors:** Paweł Nachulewicz, Anna Golonka, Tomasz Żądkowski, Paweł Osemlak, Joanna Nużyńska-Flak, Agnieszka Brodzisz, Elżbieta Pac-Kożuchowska

**Affiliations:** a Clinic of Pediatric Surgery and Traumatology; b Clinic of Pediatrics Hematology and Oncology; c Department of Pediatric Radiology; d Clinic of Pediatrics, Medical University of Lublin, Lublin, Poland.

**Keywords:** chylothorax, thoracic duct closure, thoracoscopy

## Abstract

**Background:**

We report a 16-year-old patient with a massive left-sided chylothorax after chemotherapy due to mixed germinal tumor of the testis with massive metastases located in the retroperitoneal space and posterior mediastinum. Chemotherapy resolved the metastases in the mediastinum but evoked a huge pleural effusion in the left pleural cavity, requiring surgical intervention.

Left-sided access was used. The 5-mm camera and 3 5-mm working ports were inserted. The parietal pleura was incised and the esophagus located and protected. Behind the esophagus, the thoracic duct and concomitant tissue were clipped with titanium clips, and additionally, thrombin glue was used. Stopping of the lymph leakage was observed during surgery. A local argon pleurodesis was used to finish the procedure. The thoracic tube was removed on the third postoperative day.

**Conclusion:**

Left-side access may be a good alternative in the left-sided chylothorax, but the crucial points are location and protection of the esophagus during the procedure, which is also the landmark that allows for locating the thoracic duct.

## 1 Introduction

Chylothorax is associated with high morbidity and mortality due to drainage of a large amount of lymphatic fluid. Accumulation of chyle in the pleural cavity may be either a congenital condition or the result of acquired diseases. Conservative management associated with withdrawal of enteral nutrition or administration of analogs of sandostatin requires a long treatment duration, and in many cases, it is noneffective. Surgical management, like clipping the thoracic duct or pleurodesis of the pleural cavity, is a good option, especially when performed thoracoscopically.^[1,2]^ Minimally invasive surgical access is well tolerated by patients and produces effective results with a quick resolution of symptoms. Right thoracoscopy, in almost all reported cases, is the procedure of choice because of direct access to the thoracic duct. However, in the case of a left-sided chylothorax, there is a connection with the opening of the contralateral pleural space. We present access to the thoracic duct via left thoracoscopy, which, in our opinion, may be a good alternative in the case of a left-side chylothorax.

## 2 Methods

**Figure 1:**
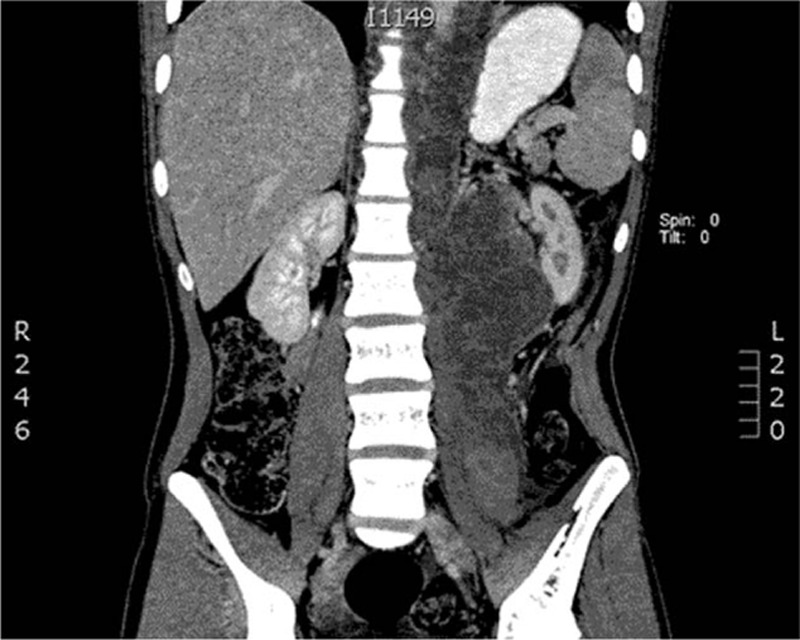
Computed tomography—massive metastases in retroperitoneal space and posterior mediastinum.

**Figure 2:**
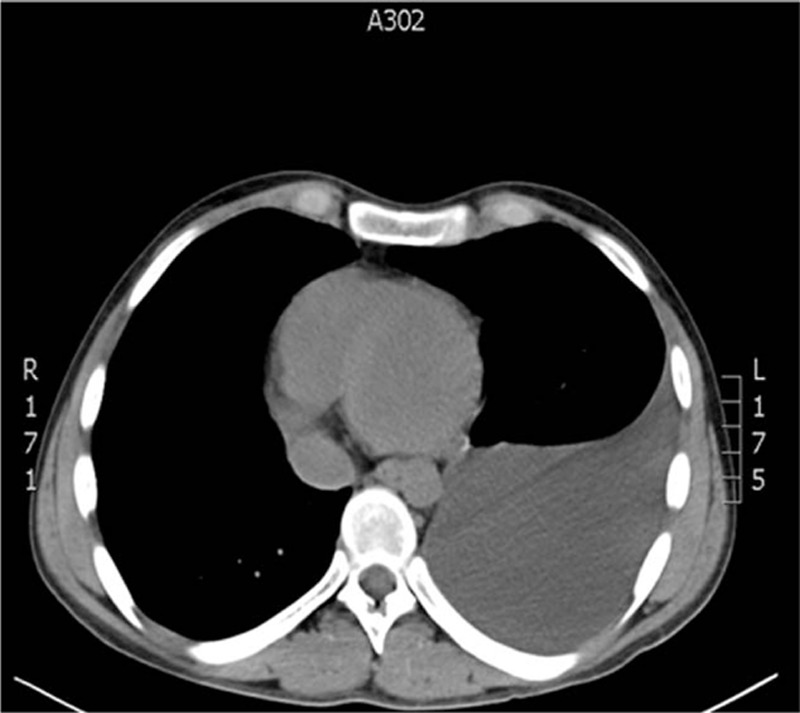
Control computed tomography after chemotherapy—massive pleural effusion in left pleural space.

**Figure 3:**
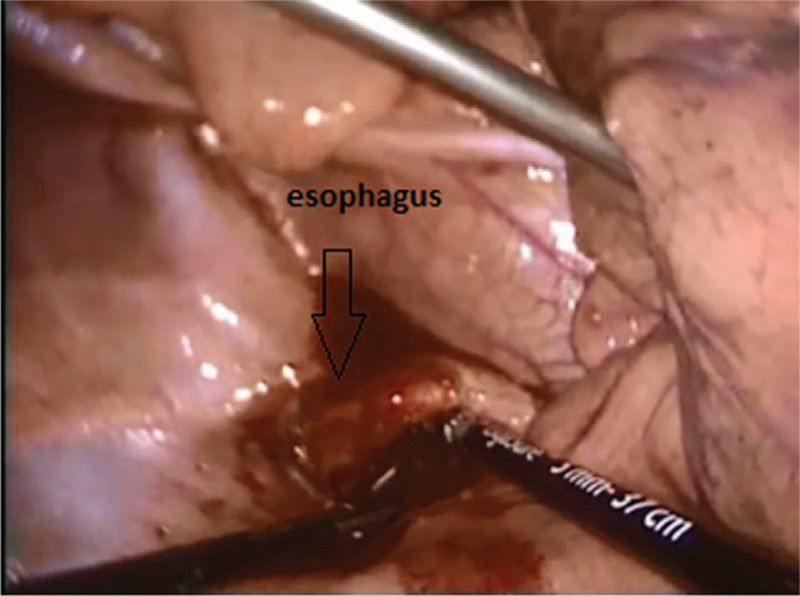
Localization of the esophagus.

**Figure 4:**
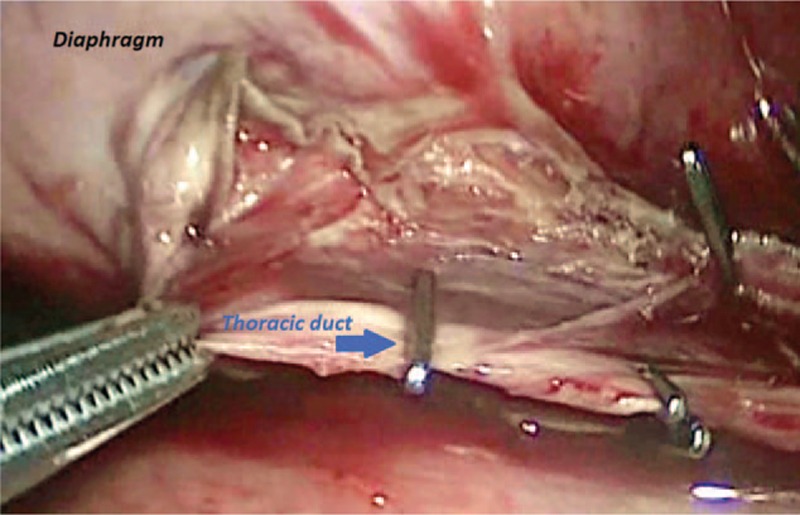
Clipped thoracic duct.

**Figure 5:**
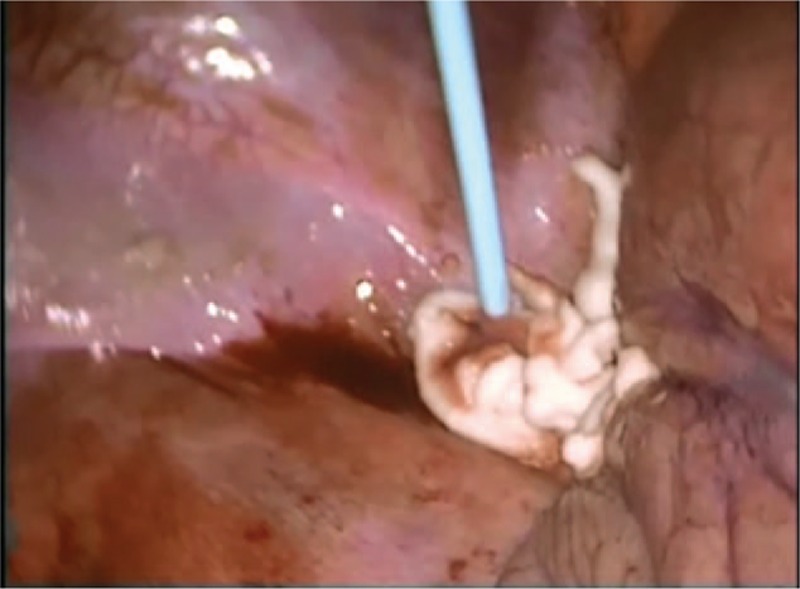
Administration of thrombin glue.

The study had informed consent of the patient accepted by Medical University of Lublin, Poland. A 17-year-old patient was admitted to the surgery unit after orchiectomy and chemotherapy for a germ cell tumor of the left testis and massive metastases located in the retroperitoneal space and posterior mediastinum (Fig. 1). A control computed tomography revealed huge remnant masses in the retroperitoneal space and resolution of the metastasis in the posterior mediastinum, but a large number of pleural effusions were observed in the left pleural cavity (Fig. 2). A thoracic tube that was inserted drained about 1.5 L of milk-stained fluid daily. The concentration of triglycerides was >110 mg/dL. Instructions of nothing-by-mouth and the inclusion of sandostatin in the treatment regime did not diminish the quantity of drained fluid during a 1-week course of conservative treatment. The left-sided thoracoscopic procedure was undertaken. The patient was intubated with a double-lumen endotracheal tube, and the lung was collapsed with the use of carbon dioxide administration at a pressure of 6 to 8 mm Hg. The 5-mm 30° camera was inserted in the fifth intercostal space, and 3 5-mm working ports were located in the seventh and ninth intercostal spaces. The parietal pleura that was medial to the aorta was incised, and the esophagus was positioned and protected (Fig. 3). Behind the esophagus, the thoracic duct and lymphatic tissue were clipped with multiple titanium clips (Fig. 4). The cessation of fluid leakage was observed during surgery. Administration of thrombin glue (SURGIFLO, Ethicon-Johnson-Johnson, Poland Sp.zoo) (Fig. 5) and regional argon pleurodesis were used to finish the procedure. The thoracic tube was left in place for 3 days postsurgery, and no drainage of fluid was observed. Five days after the thoracoscopy, the residual masses located in the retroperitoneal space were excised. The patient finished treatment 3 years ago and is now in a good general condition without any signs of recurrent disease, both in the abdomen and in the chest.

## 3 Discussion

Chylothorax is associated with significant morbidity and mortality associated with the loss of a great amount of lipids, proteins, and electrolytes. This results in deep metabolic disturbances. In addition, depletion of lymphocytes and hypogammaglobulinemia increases the risk of secondary infections.^[3]^

Causes of hydrothorax include congenital disorders, especially in small children, or acquired diseases like iatrogenic disruption of the thoracic duct during cardiac and esophageal surgery. One commonly reported reason for chylothorax is traumatic fracture of the thoracic and lumbar vertebra.^[4]^ The most common cause of malignancy associated with chylothorax is lymphoma, which is present in over 70% to 75% of cases.^[5,6]^ Chylothorax associated with germ cell tumors, as was the case in our patient, has previously not been reported.

Currently, there are no fully established standards of treatment. Insertion of the thoracic tube, an order of nothing-by-mouth, or a low-fat formula diet that replaces long-chain fatty acids with medium-chain fatty acids are usually the first-line treatments. Administration of octreotide or other analogs of sandostatin complement the conservative therapy.^[7]^ Recently, Yu et al^[8]^ reported the efficacy of etilefrine in the management of chylothorax in patients after esophagostomy where excision of the thoracic duct may exclude its effective ligation. Before the era of tharocoscopy, that treatment, albeit a long-duration therapy, was a good alternative to thoracotomy. Current results show that minimally invasive thoracoscopic closure of the thoracic duct is a safe and very effective method of treatment, and the procedure is well tolerated by patients.

In almost all reported cases, access to the thoracic duct occurs via the right pleural space because of anatomical conditions.^[9]^ The vena azygos is a good anatomical landmark that allows for quick and precise localization of the thoracic duct. Of course it is the best surgical option in right chylothorax, but in left-sided chylothorax, there is a connection with the opening of the posterior mediastinum on the opposite side, which may result in bilateral leakage if the procedure is effective. In our patient, the left chylothorax was connected with resolution of the germ cell metastases after chemotherapy, so we decided to perform thoracoscopy through the left pleural space. In this procedure, access to the thoracic duct is located on the right side of the esophagus, so the crucial point is its location. A gastric tube inserted into the esophagus allowed us to quickly, and with certainty, localize and protect the esophagus from iatrogenic injury. On the right side of the thoracic duct lies the azygos vein, so preparation has to be very careful. However, in our patient, we did not see it because we located the thoracic duct very quickly behind the esophagus. The thoracic duct was clipped, but also we clipped surrounding tissue because we did not directly see the place of leakage. In addition, there was the possibility that anatomical thoracic duct anomalies might be present (additional thoracic duct). Additional procedures, like administration of fibrin glue and pleurodesis, finished the procedure.

The prompt decision to perform thoracoscopy is especially important in patients treated due to malignant diseases. Drainage of a huge quantity of chyle may quickly deteriorate the general condition of the patients, especially after aggressive chemotherapy. Quick resolution of symptoms in our patient allowed for the removal of residual masses from the abdomen 5 days after thoracoscopy.

## 4 Conclusion

In our opinion, left-side access may be a good alternative in left-sided chylothorax, but the crucial point is location and protection of the esophagus during the procedure, which is also the landmark that allows for locating the position of the thoracic duct.
